# Effects of guanidine acetic acid supplementation from gestation to lactation on reproductive performance, colostrum quality, blood biochemistry, and intestinal microflora diversity of sows

**DOI:** 10.3389/fvets.2024.1476328

**Published:** 2024-10-23

**Authors:** Guanglei Cong, Shuangshuang Xia, Chunxue Liu, Junbo Li, Ifen Hung

**Affiliations:** ^1^Anyou Biotechnology Group Co., Ltd., Taicang, Jiangsu, China; ^2^College of Animal Science and Technology, Nanjing Agriculture University, Nanjing, Jiangsu, China

**Keywords:** pig, guanidine acetic acid, immune performance, reproductive efficiency, colostrum amino acid, nitric oxide

## Abstract

This experiment aimed to study the effects of guanidine acetic acid (GAA) on reproductive performance, lactation performance and blood biochemical indices of sows, as well as the performance of offspring piglets. A total of 20 sows (Landrace × Yorkshire, parity 4) were used. Half of the sows in each parity were fed a control diet (CG; basic diet, *n* = 10) or GAA diet (basic diet +1 g/kg GAA, *n* = 10) from the 85th day of gestation until weaning. The study results are presented as follows: Supplementation of GAA from late gestation to lactation did not adversely affect sow feed intake, backfat thickness, or blood routine indexes (*p* > 0.05). GAA supplementation showed a tendency to increase the number of healthy piglets and their birth activity (*p* = 0.06; *p* = 0.08), while significantly increasing the IUGR score of piglets (*p* < 0.05). GAA supplementation significantly increased colostrum protein content (*p* < 0.05) and tended to increase daily milk yield in sows (*p* = 0.07). GAA supplementation increased the level of immunoglobulin A in sow colostrum (*p* < 0.05) and showed a tendency to increase proline content (*p* = 0.10). GAA supplementation significantly decreased triglyceride content in sow cord blood (*p* < 0.05), with no significant effects observed on HDL-C, LDL-C, TC, and GLU (*p* > 0.05). GAA supplementation significantly increased eNOS levels in sow cord blood (*p* < 0.05), while showing no significant effects on IL-6 and IL-10 (*p* > 0.05). GAA supplementation did not significantly affect the *α* diversity of sow intestinal flora (ACE, Shannon, Chao1, Simpson, observed_otus, pielou_e, and good_cover), but PCoA analysis revealed differences in intestinal flora structure between groups. Additionally, GAA decreased the relative abundance of *Sarciha* and *unidentified_ruminococcaceae* and increased the relative abundance of *Lactobacillus*, *Parabacteroides*, and *Pedobacter* in the gut. GAA boosts nitric oxide synthase in sows’ umbilical cord blood, enhancing placental blood vessel development. This improves piglet health and vitality, increases beneficial gut bacteria (*Lactobacillus*, *Parabacteroides*, *Pedobacter*), and raises colostrum protein levels and lactation volume, leading to better piglet growth and performance.

## 1 Introduction

Guanidine acetic acid (GAA) is indispensable for creatine synthesis in vertebrates and plays a pivotal role in the energy metabolism of somatic cells in animals (1, 2). L-arginine glycine amidinotransferase catalyzes the conversion of arginine and glycine into GAA in the kidneys, which is subsequently transported via the bloodstream to the liver. Here, it reacts with n-dimethyltransferase and S-adenosylmethionine to produce creatine (3, 4). Creatine serves as a vital energy reservoir in animal tissues, rapidly converting creatine phosphate into ATP.

In recent years, the reduction in animal protein usage in Chinese livestock feed has heightened concerns over creatine loss during feed processing and heat treatment. This has led to increased interest in exogenous creatine supplementation, despite its costliness, instability, and potential inhibition of endogenous creatine synthesis (5, 6). Conversely, GAA exhibits stability and superior effectiveness compared to creatine in enhancing creatine levels in animals (7), making it a preferred option for creatine supplementation.

Studies have explored the supplementation of GAA in weaned piglets and finishing pigs, revealing that 0.12% GAA enhances average daily gain (ADG) and feed efficiency (Gratio) throughout the growth phase, increases lean meat yield, and reduces backfat thickness (8). He et al. (9) reported improvements in growth performance with 300 mg/kg of GAA, attributed to elevated creatine and ATP levels in tissues. Furthermore, supplementation with 2.4 g/kg of GAA during the nursery stage improves feed conversion efficiency and upregulates mRNA expressions of mTOR and AMPK in skeletal muscle (10). Supplementation with 0.06% GAA during the late fattening stages increases ADG and lean meat percentage by influencing muscle development through modulation of myoblast gene expression and muscle fiber characteristics (11). Additionally, supplementation around 86 kg body weight does not enhance growth performance but increases levels of free amino acids, their metabolites in plasma and tissues, and antioxidant enzymes (12, 13).

However, research on GAA’s impact in sow production remains limited and yields varied results (14, 15). Therefore, this study investigates the effects of GAA supplementation during late gestation and lactation on sow reproductive performance, colostrum composition (including immunoglobulin and amino acid content), routine cord blood parameters, nitric oxide levels in cord blood, inflammatory markers, sow intestinal flora, and growth performance of suckling piglets.

## 2 Materials and methods

The experiment was conducted at the swine experimental unit of Anyou Biotechnology Group Co., Ltd. (Nanjing, Chian). All experimental procedures were approved by the Committee on Ethics in the Use of Animals (CEUA) of the Anyou Biological Technology Group Co., LTD (No: ANS-CEUA-PJT/ PL/202309/084).

### 2.1 Experimental design, diets, and management

A total of 20 sows (Landrace × Yorkshire, parity 4) were used. Half of the sows in each parity were fed a control diet (CG; basic diet, *n* = 10) or GAA diet (basic diet +1 g/kg GAA, white powder with 98% purity, *n* = 10) from the 85th day of gestation until weaning. Detailed composition and nutrient levels of the basal diets are outlined in Table 1.

**Table 1:** Ingredients and chemical composition of basal diet (air-dried basis).

Composition, %	Gestation	Lactation	Chemical composition^b^	Gestation	Lactation
Corn	36.41	39.40	NE, Mcal/kg	2.9	3.0
Wheat middling	15.00		Crude protein, %	14.45	17.78
DDGS		5.00	Crude fat, %	5.8	6.4
Barley	9.57	15.00	Crude fiber, %	5.2	4.0
Wheat Bran	15.00	10.62	Crude ash	5.4	5.4
Full-fat rice bran		6.50	TP, %	0.70	0.63
Soybean hulls	9.50		Ca, /%	0.708	0.865
Soybean meal, 46%	7.50	14.50	Lys, %	0.86	1.19
Fish meal, 67% CP		1.00	Met, %	0.16	0.21
Limestone	0.90	1.08			
CaHPO4	0.43	0.65			
Soya-bean oil	1.69	2.25			
Premix^a^	4.00	4.00			
Total	100	100			

a Premix provided per kilogram of complete diet: vitamin A, 13, 000 IU; vitamin D3, 2020 IU; vitamin E, 40 mg; vitamin K3, 3.0 mg; vitamin B1, 3 mg; vitamin B2, 3.5 mg; vitamin B6, 2.5 mg; vitamin B12, 0.04 mg; niacin, 30 mg; vitamin C, 300 mg; folic acid, 1.5 mg; biotin, 0.3 mg; Fe (FeSO4·H2O), 100 mg; Cu (CuSO4·5H2O), 12 mg; I (KI), 0.3 mg; Se (Na2SeO3),0.2 mg; Zn (ZnSO4·H2O), 40 mg; Mn (MnSO4·H2O), 10 mg.

b NE is calculated, and other nutrient levels are measured values.

At farrowing (the farrowing day was taken as the day 0 of lactation), the number of live piglets, stillborn piglets (deformed piglets were considered stillborn), and mummified fetuses were recorded, and the birth weights of the live piglets were measured individually. Considering the same number of total live piglets which occurred per treatment, no cross-fostering was involved. Piglets were scheduled to receive plastic ear tags and supplementary iron, as well as routine procedures for tail docking, tooth clipping, and castration on the third day of age. Piglets were kept in incubators set at 22 ~ 32°C, with the temperature controlled by supplementary heating lamps. The feed (provided at 07: 30, 13: 00, and 18: 00 h) was given from 1 kg at day 1 of lactation and gradually increased by 1.0 kg per day until day 6. After that sow could access freely to feed until day 21 of lactation. During lactation, feed consumption of individual sow was recorded daily.

### 2.2 Measurement

The BF thickness and BW of unfed sows were measured on the 85th prenatal day, and the 1st and 21st postnatal days. The BF thickness was measured at the left side dorsal midline (distance 65 mm) of the 10th rib with ultrasound (Shu Shuang® Lean-Meater, China).

To calculate the average weight of piglets per litter and average weight of piglets born alive, the weight of each piglet was recorded during delivery (before eating colostrum). The number of piglets per litter (including total born, born alive, healthy piglets, Weak piglets and mummified and stillborn fetus) was recorded. Healthy piglets refer to those with a birth weight of ≥0.8 kg, while weak piglets are those with a birth weight of <0.8 kg (according to farm management standards). Record the Duration of farrowing. The duration of farrowing means the time from the birth of the first piglet to the birth of the last piglet in the litter. The daily feed intake of each sow was recorded to calculate the total feed intake and average daily feed intake.

Visual assessment of the piglet vitality scale (*VS*) was performed immediately after birth according to Baxter et al. (16). Intrauterine growth retardation (IUGR) was also measured after birth, with a score of 1 indicating normal development. A score of 2 indicates mild IUGR, with at least one IUGR. A score of 3 indicates severe IUGR in piglets with at least one IUGR display (17).

On 5 and 21-day lactation, the BW of individual piglets was weighed. During the postnatal period, piglet mortality and diarrhea were recorded daily. At the same time, feed intake of sows was recorded from day 85 gestation to day 21 lactation.

### 2.3 Sample collection

At parturition of delivery, six sows were randomly selected to collect umbilical vein blood (six umbilical veins were randomly selected in each sow). Serum samples were obtained by centrifuging the blood at 3000 r/min and 4°C for 15 min and then stored at −80°C until analysis. About 20 mL of colostrum was collected from the third and fourth pairs of hole heads on one side of the sow at 8 h after delivery, gently mixed and stored at −20°C until analysis.

#### 2.3.1 Determination of routine blood level of umbilical cord blood

The levels of white blood cell (WBC), Lymphocyte (LYM), Red blood cell (RBC), Hemoglobin (HGB), hematocrit (HCT) and platelet (PLT) in sow umbilical cord blood were determined by BH-5160 Vet animal five-classification automatic hematology analyzer (URIT, China).

#### 2.3.2 Analysis of the contents of hormones and metabolites in umbilical cord blood

The contents of Endothelial nitric oxide synthase (eNOS) were analyzed using the respective enzyme-linked immunosorbent assay (ELISA) kits (Jiangsu Meimian Industrial Co., Ltd., China) following the manufacturer’s instructions and the serum concentrations of glucose (GLU), triglyceride (TG), total cholesterol (TC), High-density lipoprotein cholesterol (HDL-C) and low-density lipoprotein cholesterol (LDL-C) were determined using Hitachi Automatic Biochemical Analyzer 3,100 (Hitachi Diagnostic Products Co., Ltd., China). The minimal detection limits for LRP and eNOS were 8 ngL/L and 0.1 μmol/L, respectively, and the intra-assay coefficient of variation (CV) of all kits was 10%, and the inter-assay CV was 12%.

The contents of Nitric Oxide (NO) was analyzed using the respective assay kits (Nanjing Jiancheng Bioengineering Institute Co., Ltd., China). All measurements are carried out in accordance with the manufacturer’s procedures.

#### 2.3.3 Analysis of the content of components in colostrum and determination of milk yield

Thawed colostrum samples were analyzed using MilkoScan™ FT3 milk analyzer (FOSS, Denmark) to assess the fat, protein, and lactose contents. The results were calculated as percentages of colostrum and milk. Evaluation of milk yields during lactation depended on the average daily gain (ADG) of individual piglets and the number of litters as per the following equation (18): milk yields = individual piglet ADG × number of litters × days of lactation × 4. From this, it concluded the average daily milk production of sows.

#### 2.3.4 Analysis of immunoglobulin content in colostrum

The contents of immunoglobulin A (IgA), IgG and IgM were analyzed using the respective enzyme-linked immunosorbent assay (ELISA) kits (Jiangsu Meimian Industrial Co., Ltd., China) following the manufacturer’s instructions. The lowest detectable levels of kit IgA, IgG and IgM were 1 μg/mL, 1.2 μg/mL and 12 μg/mL respectively, and the intra-assay coefficient of variation (CV) of all kits was 10%, and the inter-assay CV was 12%.

#### 2.3.5 Analysis of amino acid content in colostrum

According to the method proposed by Nascimento et al. (19), appropriate samples were transferred to a 50 mL hydrolysis tube, 20 mL of 6 moL/L HCL was added, and then hydrolyzed at 110°C for 24 h in an electric blast drying oven. Remove and cool, transfer to 25 mL colourimetric tube constant volume.

Accurately take 100 μL sample in 15 mL centrifuge tube, put it in a vacuum drying oven, dry it for 2 h at 60°C (dry all solvents), fill the centrifuge tube with nitrogen, and accurately add 50 μL derived reagents: Ethanol: phenyl isothiocyanate: Water: triethylamine = 7:1:1:1 (ready to use, filled with nitrogen when preparing), derived at room temperature for 30 min, added mobile phase A (31.815 g sodium acetate +3,880 mL water +120 mL acetonitrile), fixed volume to 0.5 mL, mixed well, over 0.45 μm organic membrane coating.

AA concentrations from colostrum was analyzed using oxidation analysis method on an 1,260 Infinity II Prime LC System (Agilent, United States) equipped with an RP-C18 SHISEIDO (250 mm length, 4.6 mm diameter, 5 mm particle size).

#### 2.3.6 Analysis of immune level in umbilical cord blood

The contents of interleukin-6 (IL6) and IL-10 were analyzed using the respective enzyme-linked immunosorbent assay (ELISA) kits (Jiangsu Meimian Industrial Co., Ltd., China) following the manufacturer’s instructions. The lowest detectable levels of kit IL-6 and IL-10 were 50 ng/L and 8 ng/L, respectively, and the intra-assay coefficient of variation (CV) of all kits was 10%, and the inter-assay CV was 12%.

#### 2.3.7 Analysis of intestinal flora diversity

We used DNA Kit (DP328, Tiangen Biotechnology Co., Ltd.) to extract the total genomic DNA. The integrity and concentration of RNA were detected by NanoDrop ND 2000 (Thermo, United States). According to the target fragment, PCR amplification on the V3-V4 region of 16 S rDNA, 341F-(5′-CCTAYGGGRBGCASCAG-3′), and 806R-(5′-GGACTACNNGGGTATCTAAT-3′). We then used 1.5% agarose gel electrophoresis to extract PCR products of 400–450 bp fragments, purified by GeneJET Gel Extraction Kit (Thermo, United States). The library was established by Ion Plus Fragment Library Kit (Thermo, United States). After Qubit quantification and library testing, each replicate 16 S rDNA was pooled and paired-end sequenced on IonS5TMXL sequencing platforms (Novogene Biotechnology Co., Ltd., China).

Based on IonS5TMXL sequencing platforms, the raw tags quality was filtered by FLASH (V1.2.7) and effective tags extracted. All effective tags were clustered into operational taxonomic units (OTUs) with 97% homology similarity by Uparse (V7.0.1001), using SSUrRNA database to annotate these sequences. The alpha diversity and beta diversity were analyzed based on OTUs levels.

### 2.4 Statistical analysis

All data were initially collated using Excel 2021 and analyzed with SAS 9.4 (SAS Institute Inc.). Before analysis, the UNIVARIATE procedure was used to check for outliers and assess normality with a 95% confidence interval. Data conforming to or approximating a normal distribution underwent an independent samples T-test; when variance homogeneity was not satisfied, the Satterthwaite adjustment was applied. For data not meeting normality assumptions, the Mann–Whitney U test was employed. The reproductive performance of sows was analyzed using sows and their litter sizes as the experimental unit. For other data, relevant samples from randomly selected sows within each group were used as the experimental unit. Results are presented as means and standard errors of the mean (SEM). Statistical significance was determined with *p* < 0.05, and 0.05 ≤ *p* ≤ 0.10 was considered a trend.

## 3 Results

### 3.1 Backfat thickness and feed intake

As shown in Table 2, dietary supplementation of GAA exhibited no significant impact on initial backfat, delivery backfat, weaning backfat, backfat loss, feed intake during pregnancy, and feed intake during lactation (*p* > 0.05).

**Table 2:** Effect of sows’ intake of GAA from the 85th prenatal day to entire lactation period on feed intake and backfat thickness.

Item	Control group	GAA group	*p*-value
Parity, time	4	4	–
Backfat at 26 d before delivery, mm	18.13 ± 1.13	17.13 ± 0.77	0.475
Backfat at delivery, mm	18.13 ± 1.08	18.00 ± 0.60	0.921
Backfat at weaning, mm	15.00 ± 1.06	14.67 ± 0.33	0.771
Backfat loss, mm	2.88 ± 0.58	3.13 ± 0.44	0.737
ADFI of late pregnancy, kg	3.90 ± 0.10	4.04 ± 0.03	0.225
ADFI of during lactation, kg	6.74 ± 0.19	6.86 ± 0.10	0.586

### 3.2 Umbilical cord blood routine

As shown in Table 3, dietary supplementation of GAA showed no significant effects on white blood cell count (WBC), lymphocyte count (LYM), red blood cell count (RBC), hemoglobin concentration (HGB), hematocrit (HCT), and platelet count (PLT) in cord blood of sows (*p* > 0.05).

**Table 3:** Effect of sows’ intake of GAA from the 85th prenatal day to the entire lactation period on umbilical cord blood routine.

Item	Control group	GAA group	*p*-value
WBC^1^, 10^9/L	3.92 ± 0.27	4.82 ± 0.67	0.280
LYM^2^, 10^9/L	1.94 ± 0.32	2.74 ± 0.54	0.258
RBC^3^, 10^12/L	4.13 ± 0.63	3.99 ± 0.15	0.843
HGB^4^, g/L	87.50 ± 11.99	80.17 ± 6.16	0.598
HCT^5^, %	32.00 ± 4.48	28.83 ± 1.89	0.530
PLT^6^, 10^9/L	104.00 ± 26.76	129.67 ± 36.34	0.582

1 WBC, white blood cell.

2 LYM, lymphocyte.

3 RBC, red blood cell.

4 HGB, Hemoglobin.

5 HCT, hematocrit.

6 PLT, platelet.

### 3.3 Reproductive performance

As shown in Table 4, dietary supplementation of GAA significantly increased the intrauterine growth restriction (IUGR) score (*p* < 0.05). While there was a trend toward an increased number of healthy litters and enhanced piglet vigor with GAA supplementation (*p* = 0.06; *p* = 0.08), these effects did not reach statistical significance. However, supplementation with GAA did not significantly affect parameters such as total litter number, live litter number, weak litter number, stillbirth number, healthy litter weight, average weight of live litter, stillbirth rate, weak litter rate, farrowing duration, farrowing interval, and piglet evenness in sows (*p* > 0.05).

**Table 4:** Effect of sows’ intake of GAA from the 85th prenatal day to the entire lactation period on litter performance.

Item	Control group	GAA group	*p*-value
Total number of births, head	15.63 ± 2.18	16.38 ± 1.05	0.761
Litter size born alive, head	13.50 ± 1.66	15.13 ± 0.93	0.408
Healthy litter, head	10.88 ± 1.04	13.75 ± 1.00	0.064
Weak litter, head	1.14 ± 0.40	1.38 ± 0.53	0.740
Number of dead fetuses, head	1.13 ± 0.40	1.00 ± 0.38	0.823
Weak litter rate, %	6.82 ± 2.04	8.45 ± 3.31	0.693
Stillbirth rate, %	6.80 ± 2.17	5.86 ± 1.98	0.754
Average weight of healthy piglets, kg	1.21 ± 0.04	1.30 ± 0.05	0.176
Average weight of newborn piglets, kg	1.12 ± 0.07	1.25 ± 0.06	0.172
Farrowing duration, min	188.88 ± 30.00	224.00 ± 34.06	0.452
Farrowing interval, min	11.16 ± 1.58	13.51 ± 1.51	0.303
Newborn piglet vitality	2.67 ± 0.12	2.90 ± 0.03	0.082
IUGR^1^	2.57 ± 0.11^b^	2.93 ± 0.05^a^	0.015
SD^2^	0.27 ± 0.02	0.25 ± 0.02	0.569
CV^3^, %	24.49 ± 2.63	20.60 ± 1.99	0.258

1 IUGR, intrauterine growth retardation.

2 SD, standard deviation.

3 RBC, coefficient of variation.

a Means not sharing identical superscripts in the same row are significantly different (*p* < 0.05).

b Means not sharing identical superscripts in the same row are significantly different (*p* < 0.05).

### 3.4 Composition and content of colostrum

As shown in Table 5, dietary supplementation of GAA significantly increased colostrum protein percentage in sows (*p* < 0.05). Additionally, GAA supplementation boosted milk yield (*p* < 0.05) without significantly affecting colostrum fat and lactose percentages (*p* > 0.05).

**Table 5:** Effect of sows’ intake of GAA from the 85th prenatal day to the entire lactation period on colostrum components.

Item	Control group	GAA group	*p*-value
Fat, %	5.00 ± 0.59	5.89 ± 0.63	0.333
Protein, %	16.81 ± 0.84^b^	21.10 ± 1.44^a^	0.025
Lactose, %	2.86 ± 0.28	3.19 ± 0.23	0.389
Milk yield of sow, kg	11.83 ± 0.61	13.15 ± 0.22	0.074

a Means not sharing identical superscripts in the same row are significantly different (*p* < 0.05).

b Means not sharing identical superscripts in the same row are significantly different (*p* < 0.05).

Dietary GAA supplementation increased immunoglobulin A (IgA) in sow colostrum (*p* < 0.05; Table 6), with no significant effect on colostrum IgG and IgM levels (*p* > 0.05).

**Table 6:** Effect of sows’ intake of GAA from the 85th prenatal day to the entire lactation period on Immunoglobulin content of colostrum.

Item	Control group	GAA group	*p*-value
IgA^1^, μg/ml	37.78 ± 0.73^b^	44.55 ± 0.15^a^	<0.001
IgG^2^, μg/ml	412.12 ± 8.52	411.21 ± 5.16	0.929
IgM^3^, μg/ml	42.70 ± 0.68	41.39 ± 0.68	0.201

1 IgA, immunoglobulin A.

2 IgG, immunoglobulin G.

3 IgM, immunoglobulin M.

a Means not sharing identical superscripts in the same row are significantly different (*p* < 0.05).

b Means not sharing identical superscripts in the same row are significantly different (*p* < 0.05).

Dietary GAA supplementation tended to increase proline (Pro) content in the colostrum of sows (*p* = 0.10; Table 7). Dietary GAA supplementation had no significant effects on the contents of aspartic acid (Asp), glutamic acid (Glu), serine (Ser), glycine (Gly), histidine (His), arginine (Arg), threonine (Thr), alanine (Ala), tyrosine (Tyr), valine (Val), methionine (Met), isoleucine (Iso), leucine (Leu), phenylalanine (Phe) and lysine (Lys) in sow colostrum (*p* > 0.05).

**Table 7:** Effect of sows’ intake of GAA from the 85th prenatal day to the entire lactation period on amino acid content of colostrum.

Item	Control group	GAA group	*p*-value
Asp^1^, mg/L	10449.35 ± 544.62	10694.92 ± 112.52	0.748
Glu^2^, mg/L	20486.90 ± 1102.20	21637.75 ± 1410.71	0.538
Ser^3^, mg/L	7383.15 ± 406.50	7788.58 ± 83.04	0.380
Gly^4^, mg/L	4500.55 ± 307.82	4963.92 ± 109.44	0.310
His^5^, mg/L	3974.15 ± 233.60	3835.38 ± 159.84	0.657
Arg^6^, mg/L	8614.80 ± 452.72	8843.50 ± 199.93	0.725
Thr^7^, mg/L	7972.15 ± 502.76	8523.58 ± 50.45	0.443
Ala^8^, mg/L	7277.05 ± 424.77	7678.00 ± 103.11	0.509
Pro^9^, mg/L	14049.75 ± 733.47	16752.38 ± 1014.50	0.101
Tyr^10^, mg/L	6821.15 ± 447.30	7213.25 ± 52.88	0.432
Val^11^, mg/L	10653.35 ± 612.07	11281.83 ± 59.22	0.471
Met^12^, mg/L	1788.25 ± 17.02	1885.63 ± 92.01	0.370
Ile^13^, mg/L	5735.10 ± 313.36	5783.31 ± 210.68	0.908
Leu^14^, mg/L	14370.85 ± 813.02	14953.25 ± 148.00	0.612
Phe^15^, mg/L	7084.55 ± 368.28	7468.08 ± 62.02	0.360
Lys^16^, mg/L	10453.55 ± 612.86	10766.00 ± 224.47	0.721

1 Asp, aspartic acid.

2 Glu, glutamic acid.

3 Ser, serine.

4 Gly, glycine.

5 His, histidine.

6 Arg, arginine.

7 Thr, threonine.

8 Ala, alanine.

9 Pro, proline.

10 Tyr, tyrosine.

11 Val, valine.

12 Met, methionine.

13 Ile, isoleucine.

14 Leu, leucine.

15 Phe, phenylalanine.

16 Lys, lysine.

### 3.5 Serum metabolites, NO and immune performance

As shown in Table 8, dietary GAA supplementation tended to decrease serum TG levels in sow cord blood (*p* = 0.072). However, dietary GAA supplementation did not significantly affect levels of HDL-C, LDL-C, GLU, and TC in sow cord blood (*p* > 0.05).

**Table 8:** Effect of sows’ intake of GAA from the 85th prenatal day to the entire lactation period on serum metabolites.

Item	Control group	GAA group	*p*-value
HDL-C^1^, mmol/L	0.45 ± 0.06	0.33 ± 0.05	0.158
LDL-C^2^, mmol/L	0.65 ± 0.08	0.49 ± 0.07	0.181
GLU^3^, mmol/L	0.13 ± 0.002	0.13 ± 0.001	0.122
TC^4^, mmol/L	1.14 ± 0.13	0.88 ± 0.12	0.184
TG^5^, mmol/L	1.58 ± 0.16	1.15 ± 0.15	0.072

1 HDL-C, high density lipoprotein cholesterol.

2 LDL-C, low density lipoprotein cholesterol.

3 GLU, glucose.

4 TC, total cholesterol.

5 TG, triglyceride.

Dietary supplementation of GAA significantly increased the eNOS level in sow cord blood (*p* < 0.05; Table 9). However, levels of NO, IL-6, and IL-10 in sow cord blood were not significantly altered by dietary GAA supplementation (*p* > 0.05).

**Table 9:** Effect of sows’ intake of GAA from the 85th prenatal day to the entire lactation period on NO and immune performance.

Item	Control group	GAA group	*p*-value
NO^1^, μmol/L	11.81 ± 0.90	12.37 ± 0.52	0.600
eNOS^2^,μmol/L	4.51 ± 0.07^b^	5.20 ± 0.07^a^	<0.001
IL-6^3^, ng/L	1099.96 ± 11.17	1113.12 ± 19.46	0.593
IL-10^4^, ng/L	214.91 ± 2.77	215.97 ± 3.02	0.802

1 NO, nitric oxide.

2 eNOS, endothelial nitric oxide synthase.

3 IL-6, interleukin-6.

4 IL-10, interleukin-10.

a Means not sharing identical superscripts in the same row are significantly different (*p* < 0.05).

b Means not sharing identical superscripts in the same row are significantly different (*p* < 0.05).

### 3.6 Intestinal flora diversity

**Figure 1:**
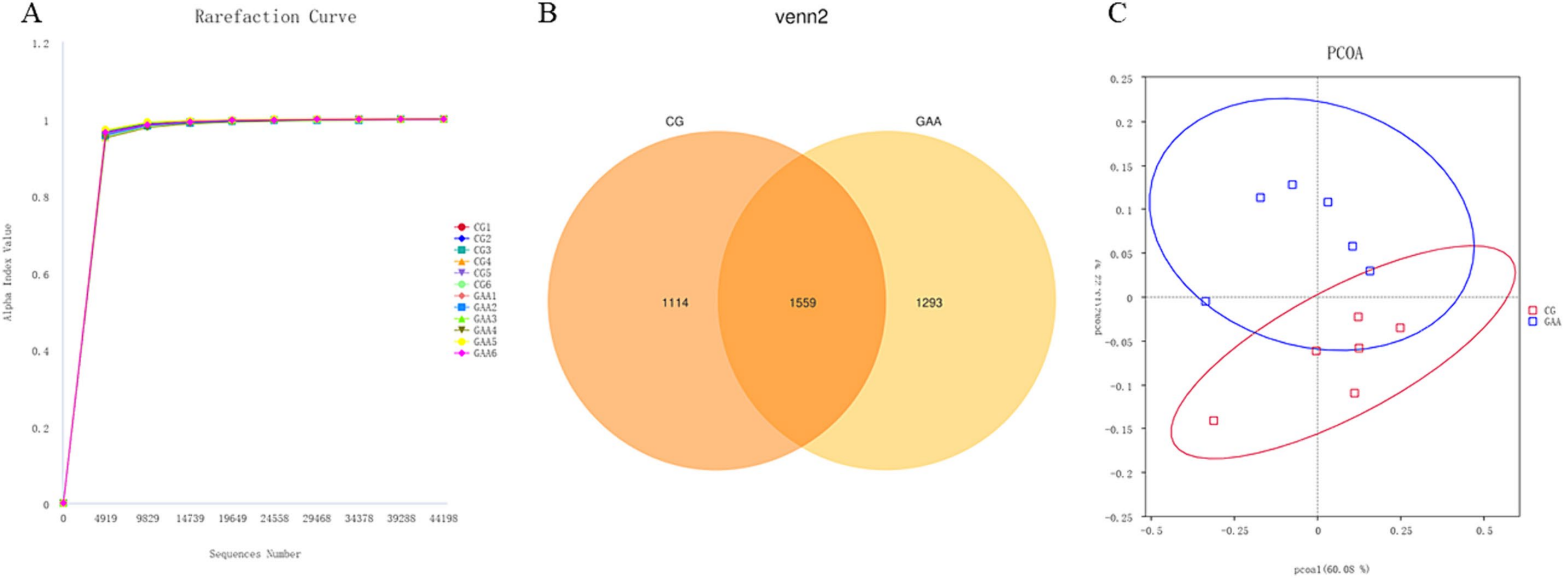
GAA dilution curve **(A)**, Venn diagram **(B)** and PCoA **(C)** analysis of intestinal flora of sows.

Based on Figure 1, the dilution curves of fecal samples from both groups of sows showed a steady upward trend, indicating thorough sample extraction. According to the Venn diagram, the control group (CG) exhibited 1,114 unique sequences, while the guanidine acetic acid group (GAA) had 1,293 unique sequences, with 1,559 sequences shared between the two groups. PCoA analysis revealed significant differences in the composition and structure of gut microbiota between the two groups.

**Figure 2:**
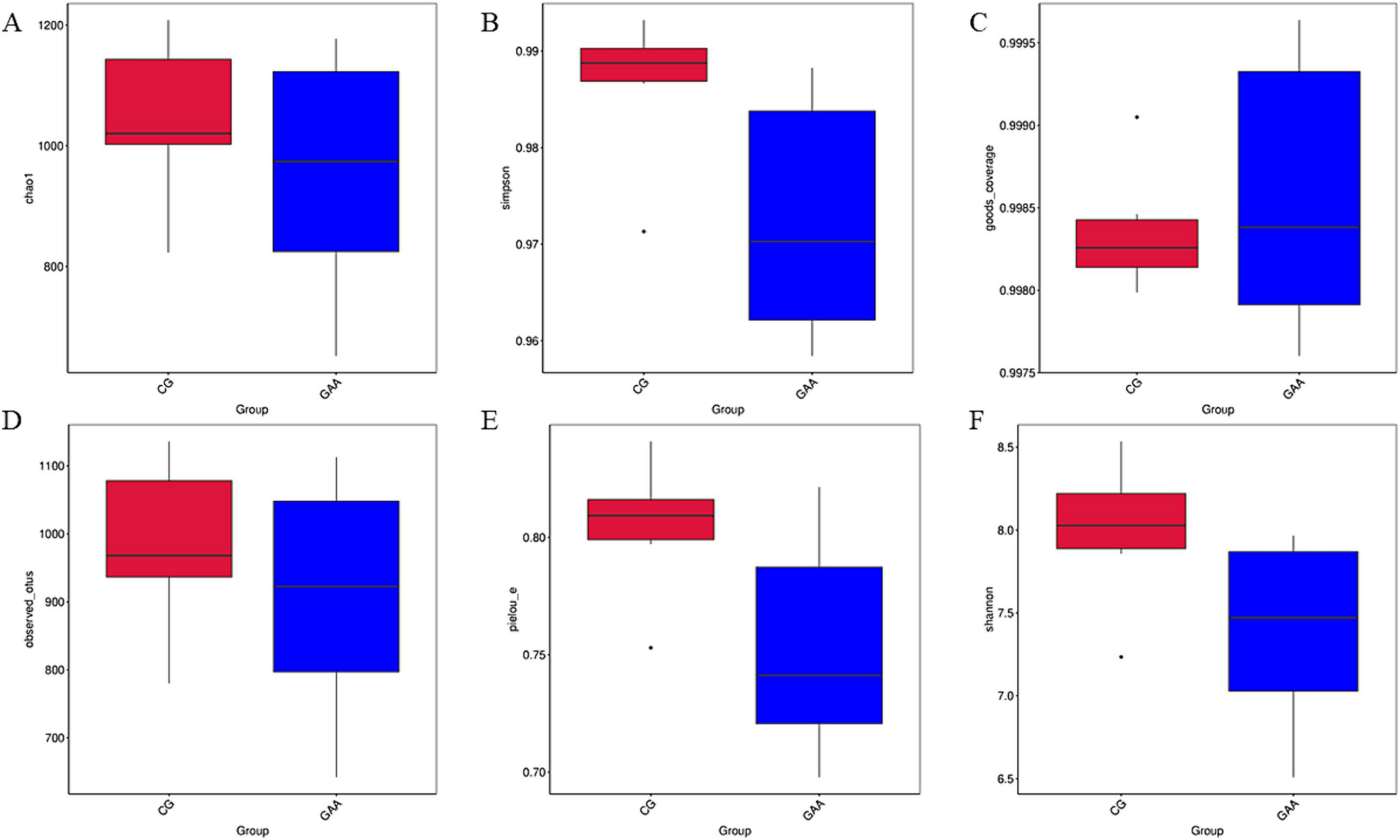
Effects of dietary GAA supplementation on *α*-diversity of intestinal flora in sows: **(A)** chao1 diversity index; **(B)** simpson diversity index; **(C)** good_coverage diversity index; **(D)** observed_otus diversity index; **(E)** pielou_e diversity index; **(F)** shannon diversity index.

As shown in Figure 2, there were no significant differences (*p* > 0.05) in *α*-diversity indices (chao1, simpson, good_coverage, observed_otus, pielou_e, and shannon) of gut microbiota between the two groups.

**Figure 3:**
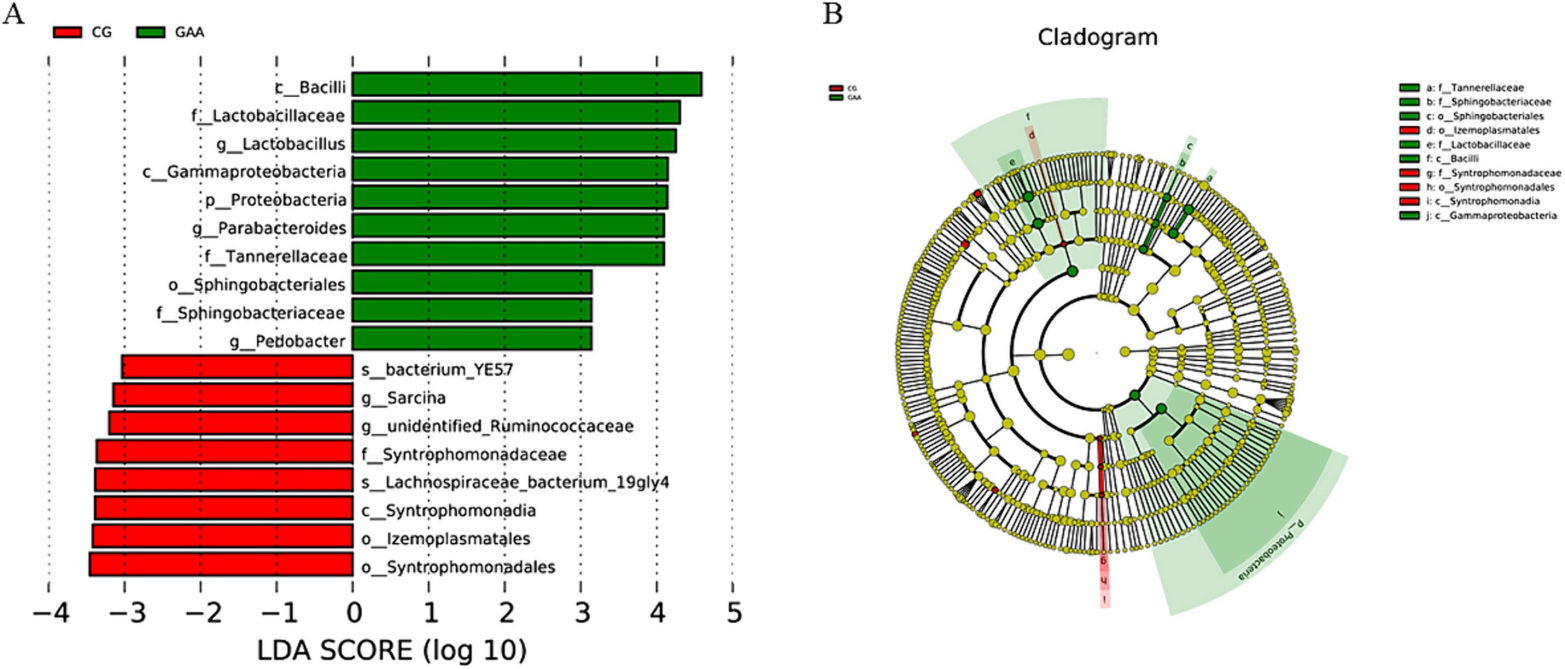
The effects of GAA on the β-diversity of intestinal flora of sows were analyzed by LEfSe; **(A)** LDA Score; **(B)** Cladogram.

LEfSe analysis in Figure 3, LDA = 3, identified *lactobacillus, Pedobacter, and Parabacteroides* as significantly enriched taxa in the gut microbiota of sows supplemented with guanidine acetic acid.

**Figure 4:**
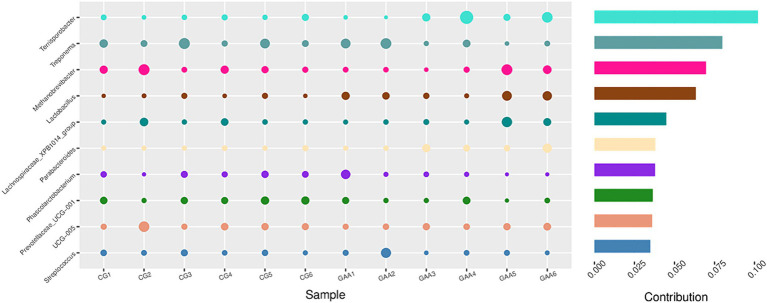
Simper analyzed the effects of GAA on the β-diversity of the intestinal flora of sows.

Furthermore, Simper analysis in Figure 4 further identified *lactobacillus and Terrisporobacter* as dominant taxa in the gut microbiota of the guanidine acetic acid group.

### 3.7 Growth performance and health of suckling piglets

As shown in Table 10, dietary supplementation with GAA resulted in a significant increase in litter weight gain of offspring piglets (*p* < 0.05). Moreover, the addition of GAA to the diet showed a tendency to increase the average daily gain (ADG) of piglets (*p* < 0 0.05). However, supplementation with GAA did not significantly affect litter size at weaning, litter weight at 5 days, litter weight at weaning, piglet diarrhea rate, or the survival of suckling piglets.

**Table 10:** Effects of GAA supplementation on growth performance and health of offspring piglets of sows from the 88th day before delivery to the whole lactation period.

Item	Control group	GAA group	*p*-value
Litter size at weaning, head	11.13 ± 0.40	11.63 ± 0.26	0.312
Litter weight at 5 d, kg	29.07 ± 2.22	26.58 ± 0.67	0.313
Litter weight at weaning, kg	70.86 ± 5.21	78.03 ± 1.06	0.217
Litter weight gain, kg	41.79 ± 3.41^b^	51.45 ± 1.07^a^	0.026
Individual piglet weight, kg	6.89 ± 0.28	6.73 ± 0.13	0.613
ADG of piglet, g	236.62 ± 10.69	261.20 ± 7.01	0.070
Piglet diarrhea rate, %	0.06 ± 0.06	0.17 ± 0.08	0.346
Survival rate, %	91.89 ± 2.98	92.23 ± 2.06	0.927

## 4 Discussion

Currently, there is limited research on the application of guanidine acetic acid (GAA) in sows. Pregnant and lactating sows have peak energy requirements, and supplementing with GAA during this period can enhance energy efficiency, thereby increasing sow and offspring productivity.

Research indicates that GAA supplementation during gestation alone can boost the number of live piglets per sow. Starting GAA supplementation in late gestation enhances the average birth weight of piglets, while supplementation during lactation increases protein and amino acid concentrations in sow colostrum (20). Creatine supplementation in the final week of gestation improves the myelin sheath of low birth weight piglets and increases the survival rate of weaker piglets, though it has no impact on sow labor duration, piglet birth intervals, or stillbirth rates (21). Adding 0.1% GAA to gilt diets enhances the number of live piglets produced, with minimal variation in birth weights per litter. Moreover, GAA enhances average daily gain (ADG), weaning weights, and daily milk yield of piglets. During lactation, GAA increases amino acid content in milk by the 7th day, particularly lysine, methionine, arginine, valine, and glutamine (15).

Our results indicate that GAA supplementation significantly increases the number of live and healthy piglets but does not affect sow farrow duration, litter intervals, stillbirth rates, feed intake, or backfat loss. This finding aligns with previous studies, suggesting that GAA’s growth-promoting effects in monogastric animals are primarily related to its role in tissue protein anabolism and improvement of energy metabolism. The synthesis of GAA involves its production in the liver and subsequent methylation in the kidneys. GAA synthesis requires the participation of two amino acids, arginine and glycine. Arginine transfers its amidino group to the amino group of glycine to produce ornithine and GAA, catalyzed by *L-arginine*: glycine amidinotransferase (AGAT). Guanidinoacetate is then methylated using a methyl group from S-adenosylmethionine (SAM), which is synthesized from methionine. This reaction, catalyzed by guanidinoacetate N-methyltransferase (GAMT), produces creatine and S-adenosylhomocysteine (SAH) (2). Consequently, dietary GAA supplementation reduces the breakdown of arginine and glycine in the body, thereby enhancing their efficacy in protein synthesis and promoting growth (22, 23). Additionally, GAA may be mediated through transport proteins such as creatine transporter (CRT/SLC6A8), taurine transporter (TauT/SLC6A6), and *γ*-aminobutyric acid transporter (SLC6A13), and is transported into target cells via passive diffusion across the plasma membrane (24), thereby improving reproductive performance in sows. Moreover, GAA’s physiological regulatory functions may include stimulating the secretion of insulin-like growth factor 1 (IGF-1), thereby enhancing growth performance (25). Furthermore, dietary GAA supplementation might boost the secretion of gamma-aminobutyric acid (GABA), which promotes the secretion of growth hormone-releasing hormone by the hypothalamus and subsequently increases growth hormone secretion by the adenohypophysis, fostering animal growth (26). However, our current study on GAA in sows has limitations regarding the mechanisms involved, warranting further investigation and validation.

In conjunction with the nitric oxide findings from our experiment, nitric oxide synthase and nitric oxide levels in sow cord blood increased by 0.7 μmol/L and 0.5 μmol/L in the GAA group, respectively. Optimal nitric oxide levels stimulate blood vessel proliferation in the placenta and umbilical cord, enhancing nutrient flow from mother to piglet and increasing piglet birth weights. This underscores why guanidine acetic acid can increase the number of healthy piglets in sows, resulting in an average weight increase of 130 g for live piglets and 90 g for healthy piglets.

During gestation, arginine deficiency can be effectively mitigated by supplementing with guanidine acetic acid (GAA). Mateo et al. (27) observed that daily supplementation of 1% arginine in lactating sows on the 7th day of lactation increased plasma arginine concentrations and insulin levels, thereby enhancing substance metabolism. This supplementation also elevated nutrient content in milk, with higher concentrations of most amino acids compared to the control group. Additionally, arginine promotes mammary gland development, improves blood circulation, affects nutrient transport, and potentially enhances lactating performance by increasing milk yield and quality (28).

Moreover, our study demonstrated that GAA supplementation increased milk protein, urea nitrogen, and total solids in sow colostrum. This suggests that GAA may influence colostrum composition through its impact on arginine levels. Further investigation into normal milk composition in sows was not conducted in this experiment, highlighting the need for future research.

The intestinal microbiota plays a crucial role in regulating physiological development and health status in pigs, as well as preventing pathogen colonization (29). While research on GAA’s effect on intestinal flora is sparse, studies on arginine have shown that intestinal bacteria utilization can lead to polyamine release in the intestine (30). Arginine also regulates amino acid utilization in intestinal bacteria cultures obtained from pig intestines (31, 32).

In our experiment, the GAA group did not affect the *α* diversity of sow intestinal flora but did show differences in *β*-diversity. LEfSe and Simper analyses identified *Lactobacillus and Terrisporobacter* as the main genera affected. Lactobacillus, a probiotic, inhibits harmful microorganisms (33), alters microbial metabolism (enzyme activity), enhances nutrient absorption (e.g., proteins, monosaccharides) (34), and stimulates immune responses (35). The Terrisporobacter genus includes beneficial bacteria like *Bacillus subtilis*, *Bacillus fungoides, and Bacillus polymyxis*, which improve animal production performance. These findings suggest that GAA alters dominant intestinal bacteria, potentially improving nutrient metabolism and absorption, thereby increasing milk protein, total solids, and urea nitrogen in sows.

GAA enters sow milk through the bloodstream and effectively increases creatine and creatine phosphate content in muscle tissue. This reduces carbohydrate, fat, and protein energy supplies while enhancing hydration to inhibit protein breakdown and promote protein and glycogen synthesis, accelerating animal growth (36). Additionally, GAA influences the hypothalamus to secrete growth hormone-releasing hormone, impacting gamma-aminobutyric acid secretion and subsequently stimulating pituitary growth hormone secretion (37). Combined with the increased milk protein levels in sow milk, this contributes to increased average daily gain (ADG) in offspring piglets to a certain extent.

## 5 Conclusion

**Figure 5:**
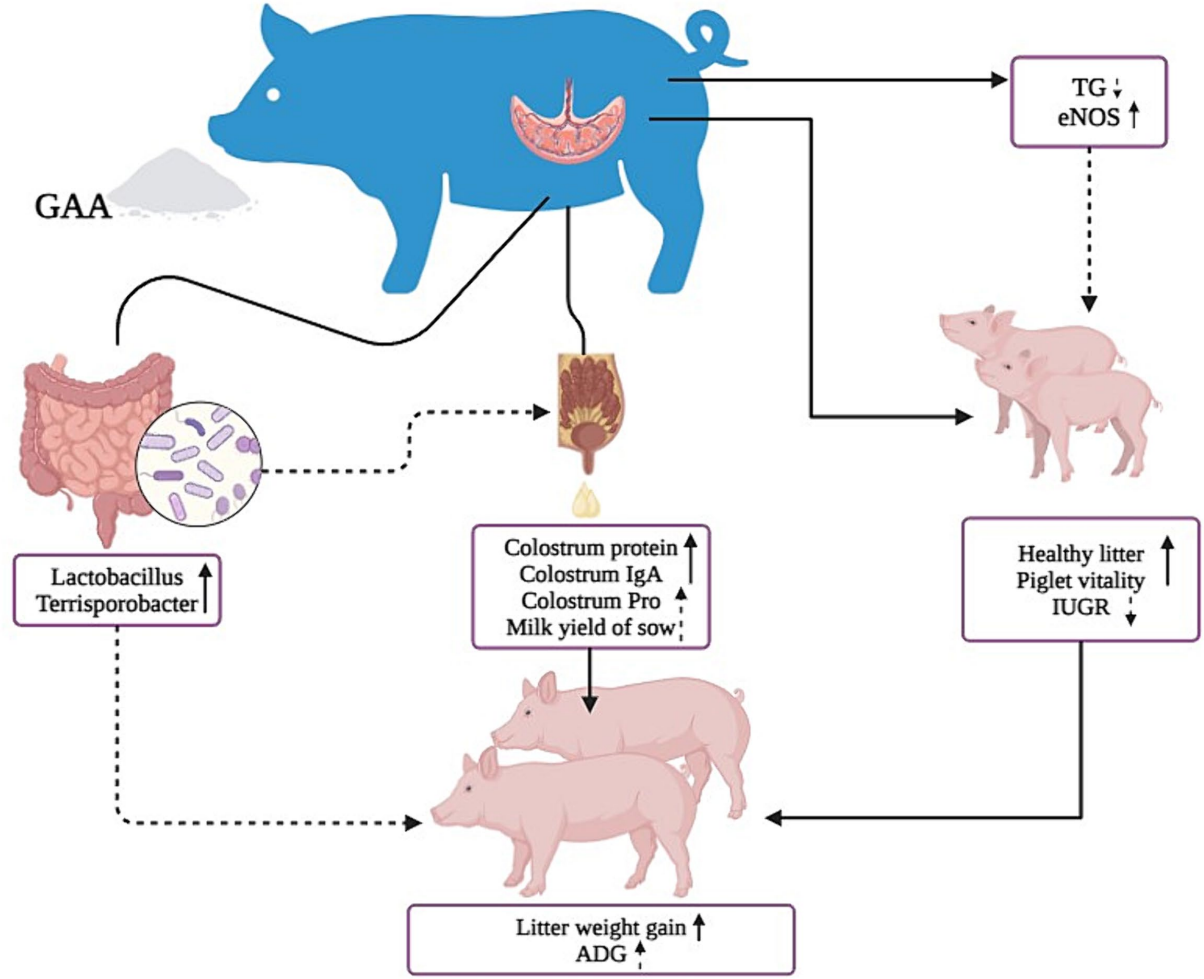
Graphical representation of the effects of GAA on sow reproductive performance, lactation performance, intestinal flora, and offspring piglet.

GAA can increase nitric oxide synthase levels in sows’ umbilical cord blood, promoting the development of placental blood vessels and piglet growth. It enhances the number of healthy piglets and their vitality, while also increasing the abundance of beneficial bacteria such as *Lactobacillus Parabacteroides*, and Pedobacter in the gut. These effects lead to higher protein levels in colostrum and increased total lactation volume, thereby improving the growth performance of offspring piglets (Figure 5).

## Data Availability

The raw data supporting the conclusions of this article will be made available by the authors, without undue reservation.
